# Hyperkalemia Management with Intravenous Insulin in Patients with Reduced Kidney Function

**DOI:** 10.3390/jcm13175103

**Published:** 2024-08-28

**Authors:** Maram A. Alzahrani, Numan A. AlAbdan, Zainab S. Alahmari, Nouf M. Alshehri, Lama H. Alotaibi, Omar A. Almohammed

**Affiliations:** 1 Pharmaceutical Care Department, King Faisal Specialist Hospital and Research Centre, Riyadh 12713, Saudi Arabia; pharmd.maram@hotmail.com; 2 Pharmaceutical Care Department, King Abdulaziz Medical City–Ministry of National Guard Health Affairs, Riyadh 11426, Saudi Arabia; aboanasalabdan@gmail.com (N.A.A.); zainab.s.alahmari@gmail.com (Z.S.A.); alashwal.nouf@gmail.com (N.M.A.); lama.h.alotaibi@gmail.com (L.H.A.); 3 King Abdullah International Medical Research Center, Riyadh 11481, Saudi Arabia; 4 Department of Clinical Pharmacy, College of Pharmacy, King Saud University, Riyadh 11451, Saudi Arabia; 5 Pharmacoeconomics Research Unit, College of Pharmacy, King Saud University, Riyadh 11451, Saudi Arabia

**Keywords:** hyperkalemia, insulin, hypoglycemia, estimated glomerular filtration rate, reduced kidney function, hemodialysis

## Abstract

**Background**: Insufficient kidney function increases the risk of hyperkalemia and hypoglycemia, particularly in hemodialysis-dependent patients. Hypoglycemia is a common complication of insulin-based hyperkalemia treatment. This study aims to evaluate the efficacy and safety of hyperkalemia treatment in hemodialysis-dependent and -non-dependent patients and identify risk factors associated with hypoglycemia. **Methods**: A retrospective observational cohort study was conducted to assess the efficacy and safety of hyperkalemia treatment including patients with reduced kidney function and hyperkalemia treated with intravenous insulin. The decline rate of potassium and glucose levels were compared between hemodialysis-dependent and non-dependent patients. In addition, univariate and multivariable logistic regression analyses were performed to identify risk factors associated with hypoglycemia. **Results**: A total of 172 patients with hyperkalemia and reduced kidney function were included. The steepest reduction of serum potassium levels happened within the first 6 h after insulin administration, at 1.1 and 0.9 mmol/L for hemodialysis-dependent and non-dependent patients, respectively. The incidence of hypoglycemia was 18%, and no significant difference was found between cohorts. Hemodialysis-dependent patients were more likely to be readmitted within one month with hyperkalemia, while all-cause ICU admission was more likely for non-dependent patients. Older patients, and those who had heart failure or received a second dose of insulin to treat hyperkalemia, were more likely to experience hypoglycemia. **Conclusions**: Monitoring blood glucose levels following insulin administration is essential given the complexity of patients’ factors associated with hypoglycemia resulting from hyperkalemia treatment in patients with insufficient kidney function.

## 1. Introduction

Reduced kidney function is a predisposing factor for hyperkalemia (serum potassium level > 5.5 mmol/L), primarily attributed to the inability of the impaired kidney to effectively regulate potassium levels. However, increased potassium intake, certain medications, and comorbidities add to the risk as well [1]. Furthermore, hyperkalemia risk seems to increase after starting hemodialysis [2,3], and considerable morbidity and mortality is linked to hyperkalemia [4,5]. That said, hyperkalemia is a critical condition that can lead to muscle weakness causing paralysis or respiratory failure, severe cardiac arrhythmias, cardiac arrest, and overall increased mortality [6,7,8]. 

Clinical guidelines recommend treatment for hyperkalemia should include the administration of intravenous (IV) insulin in doses ranging from 5 to 10 units, along with 25 to 50 g of dextrose [9,10]. Insulin reduces serum potassium levels by shifting it into the cells—this promptly restores the electrolyte balance [11]. In hemodialysis-dependent patients, this may contribute to hyperkalemia rebound after dialysis [12]. Hypoglycemia is a common complication after the administration of insulin [13], which is a concern in patients with reduced kidney function [14,15,16,17]. The incidence of hypoglycemia in this context varies greatly in the literature—between 6.1% and 75%—but this range is distorted by an outlier study with a small sample size of 12 patients [15,17,18,19,20]. 

Many factors make reduced kidney function an independent risk factor for hypoglycemia [21]. The kidneys’ inability to contribute to gluconeogenesis as well as the delay in the clearance of insulin and other antidiabetic medications may prolong their effect [22,23]. Hemodialysis-dependent patients are at risk of hypoglycemia because of the prolonged effect of insulin, which can outlast the duration of dextrose in the bloodstream [11]. Moreover, the use of glucose-free dialysis solutions increases the risk of hemodialysis-induced hypoglycemia in dependent patients [24,25].

Given the complex interplay between hemodialysis, insulin-based hyperkalemia treatment, and the risk of hypoglycemia, further research is needed to evaluate the outcomes of hyperkalemia management with insulin in patients with reduced kidney function, especially between hemodialysis-dependent and non-dependent patients. As limited research was conducted to address the outcomes and complications of acute hyperkalemia management between the aforementioned cohorts, this study aims to address this gap in the literature. The primary objective was to assess the effectiveness of insulin in lowering potassium levels and compare the extent of the reduction in certain time frames, in addition to investigating the incidence of hypoglycemia as a complication of insulin therapy between hemodialysis-dependent and non-dependent patients. 

This study also intends to compare the risk of other outcomes like all-cause ICU admission and all-cause and hyperkalemia-caused hospital readmission within one month between cohorts. It also aims to identify the risk factors associated with hypoglycemia by comparing patients’ demographics, comorbidities, baseline lab results, and the use of medications that increase potassium levels as well as the therapeutic interventions received. Lastly, understanding therapeutic outcomes and the complications associated with hyperkalemia treatment strategies is crucial for guiding clinical decision-making and optimizing patient care. Thus, comparing potassium and glucose levels after insulin administration can provide insights into the impact of hemodialysis and other factors on its efficacy and safety when treating patients with hyperkalemia.

## 2. Materials and Methods

### 2.1. Study Design, Setting, Subjects, and Ethical Approval

A retrospective observational cohort study was conducted to assess the effectiveness and safety of intravenous insulin in lowering potassium levels and restoring electrolyte balance in patients with hyperkalemia and reduced kidney function, especially between hemodialysis-dependent and non-dependent patients. The data for the study were retrospectively collected from King Abdulaziz Medical City (KAMC) in Riyadh, Saudi Arabia. Patients were deemed eligible to be included if they had AKI or CKD (estimated glomerular filtration rate [eGFR] < 60 mL/min/1.73m^2^ for over three months) [26,27], developed hyperkalemia (potassium level > 5.5 mmol/L) [28,29], and were treated with 10 units of intravenous insulin for the management of hyperkalemia within 6 h of collecting the potassium level [30]. Patients receiving insulin doses of more than 10 units, or more than a single dose of insulin within 6 h, for hyperkalemia management were excluded; this was due to the increased risk of hypoglycemia in these patients compared to the rest in the study. Also, patients who had hyperkalemia occurring due to the most serious acute complications of diabetes (i.e., diabetic ketoacidosis and hyperosmolar hyperglycemic state) were excluded from this study because their management approach would be different. Before commencing this research study, ethical approval was obtained from the Institutional Review Board (IRB) of the King Abdullah International Medical Research Center (KAIMRC) (Reference # NRC22R/082/02).

### 2.2. Data Collection and Management

A standard data collection form was developed on Microsoft Excel for the extraction of patients’ data from electronic medical records. For patients with reduced kidney function with/without hemodialysis, data regarding the sociodemographic characteristics (age and sex), anthropometric measures (body mass index; BMI), chronic comorbidities (diabetes mellitus, hypertension, heart failure, and ischemic heart disease), clinical and laboratory data (blood glucose, serum creatinine, eGFR, and serum potassium), possible causes of hyperkalemia (diseases and drugs), and clinical management of hyperkalemia (insulin, calcium, *β*-agonist, sodium bicarbonate, diuretics, and hemodialysis) were recorded. The eGFR was calculated using the Chronic Kidney Disease Epidemiology Collaboration (CKD-EPI) equation [31]. Hypoglycemia was described as blood glucose levels ≤ 3.9 mmol/L (70 mg/dL) [16].

### 2.3. Study Outcomes

The primary study outcome was to determine and compare the outcome of hyperkalemia management with insulin in patients with reduced kidney function, namely between hemodialysis-dependent and -non-dependent patients. The secondary study outcomes were to measure the incidence of hypoglycemia over time in patients with reduced kidney function (including hemodialysis-dependent and non-dependent patients) and identify factors associated with hypoglycemia.

### 2.4. Statistical Analysis

For descriptive analysis, continuous data were reported as mean ± standard deviation (SD), while categorical data were reported as frequency with percentages. To compare the continuous data variables between the two cohorts (hemodialysis-dependent and non-dependent patients with reduced kidney function), an independent sample t-test was used. The categorical data variables were compared between the groups using the chi-square or Fisher’s exact tests. Univariate and multivariable logistic regression analyses were performed to identify the risk factors associated with hypoglycemia in patients with reduced kidney function. *p* < 0.05 was considered statistically significant for all the statistical tests. All data management and analyses were performed using SAS software, version 9.4 (SAS Institute Inc., Cary, NC, USA). 

## 3. Results

### 3.1. Baseline Characteristics of Study Subjects

A total of 172 patients with reduced kidney function and hyperkalemia were included in this study. A total of 75 patients (43.6%) were on hemodialysis therapy. Higher serum creatinine (565.0 ± 207.5 vs. 146.4 ± 94.0 µmol/L, *p* < 0.001) and potassium levels (6.7 ± 0.8 vs. 6.5 ± 0.5 mmol/L, *p* = 0.049) were observed in hemodialysis-dependent patients compared to non-dependent patients. Despite hemodialysis-dependent patients being, on average, younger than their counterparts who were hemodialysis-non-dependent (61.3 ± 16.1 vs. 68.1 ± 13.6 years, *p* = 0.003), they were less likely to have heart failure as a comorbidity and less likely to be on medications that increase potassium levels, i.e., angiotensin-converting enzyme inhibitors (ACE-Is), angiotensin receptor blockers (ARBs), or potassium-sparing diuretics, as shown in Table 1. 

### 3.2. Standard Management of Hyperkalemia

All patients received 10 units of intravenous insulin for the management of hyperkalemia. Regardless of the patient group, the majority received β-agonist (83.7%) and calcium (53.5%). However, the correction of hyperkalemia with dialysis was more common among hemodialysis-dependent patients compared to non-dependent patients (37.3% vs. 4.1%; *p* < 0.001), but these patients were less likely to be treated with sodium bicarbonate (13.3% vs. 42.3%; *p* < 0.001) or diuretics (5.3% vs. 30.9%; *p* < 0.001) compared to non-dependent patients, as shown in Table 2.

### 3.3. Hypoglycemia and Other Outcomes for Hyperkalemia Management 

**Figure 1:**
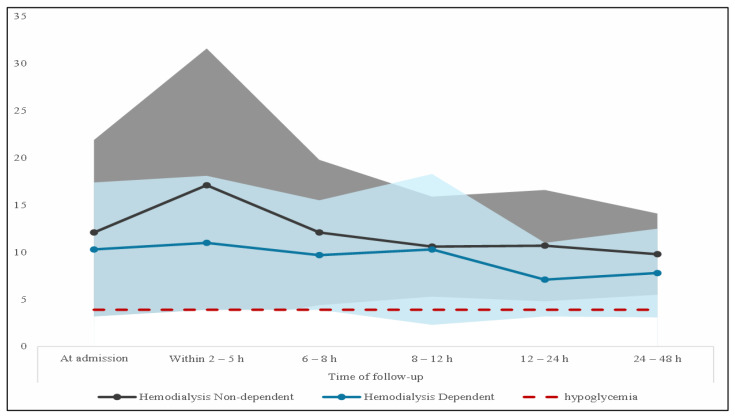
Glucose level over time (mmol/L) in each group. The area in light blue and dark gray represents the range for the glucose level at each follow-up for hemodialysis dependent and non-dependent patients, respectively.

Overall, a total of 31 patients (18.0%) experienced hypoglycemia (blood glucose levels ≤ 3.9 mmol/L). A higher percentage of hemodialysis-dependent patients experienced hypoglycemia within the first 24 h after insulin administration, but the difference between the groups was not statistically significant (20.0% vs. 16.5%, *p* = 0.553), despite them having a lower blood glucose level at admission (10.3 ± 7.1 vs. 12.1 ± 9.8 mmol/L) and for the following 48 h. They also did not experience the glucose spike within the first 2 to 5 h, nor its steady decline thereafter, as was the case with their hemodialysis-non-dependent counterparts. Instead, their blood glucose dropped drastically in the second 12 h after initiating the hyperkalemia treatment, which was the period when many patients on hemodialysis experienced hypoglycemia, as shown in Figure 1.

**Figure 2:**
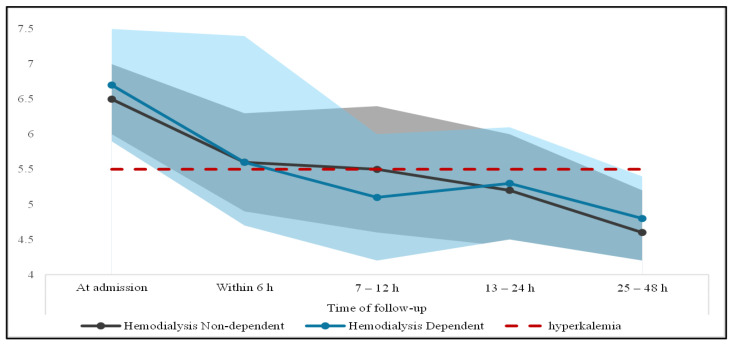
Potassium level over time (mmol/L) in each group. The area in light blue and dark gray represents the range for the glucose level at each follow-up for hemodialysis dependent and non-dependent patients, respectively.

Moreover, within the first 6 h, serum potassium levels had the steepest reduction, at 1.1 and 0.9 mmol/L for hemodialysis-dependent and non-dependent patients, respectively. Both cohorts continued to have a steady decline in potassium levels for the first 12 h following the initiation of hyperkalemia treatment. However, in the second 12 h, patients on hemodialysis had an average increase of 0.2 mmol/L, while their counterparts continued the steady decline, as shown in Figure 2. Invariably, hemodialysis-dependent patients were more likely to be readmitted within one month with hyperkalemia (9.3% vs. 2.1%, *p* = 0.042). On the other hand, admission to the intensive care unit (ICU) for any cause was more frequent in hemodialysis-non-dependent patients (21.6% vs. 6.7%, *p* = 0.006), as shown in Table 3.

### 3.4. Risk Factors Associated with Hypoglycemia

While the risk of hypoglycemia was nearly the same for both cohorts, other factors were associated with hypoglycemia in the univariate and multivariable logistic regression analyses, including age, heart failure, and receiving dialysis or a second dose of insulin. Patients who experienced hypoglycemia tended to be slightly older (68.9 ± 11.3 vs. 64.3 ± 15.7 years). Patients with heart failure (OR 2.43, 95%CI 1.03–5.69) and patients who received dialysis (OR 2.58, 95%CI 1.07–6.21) were twice as likely to experience hypoglycemia. Moreover, the most indicative risk factor was receiving a second dose of insulin, as patients who had hypoglycemia had three times the odds of having received a second dose of insulin (OR 3.11, 95%CI 1.38–7.00), as shown in Table 4. In the multivariable logistic regression analysis, age (OR 1.04, 95%CI 1.00–1.07), having heart failure (OR 2.80, 95%CI 1.09–7.19), and receiving a second dose of insulin (OR 4.14, 95%CI 1.68–10.21) were found to be associated with an increased risk of hypoglycemia in patients with reduced kidney function and hyperkalemia (Table 4).

### 3.5. Sub-Analysis for Patients Who Received a Second Dose of Insulin

On the other hand, fewer hemodialysis-dependent patients received a second dose of insulin—around one-third—compared to almost half of the non-dependent patients (32.0% vs. 49.5%; *p* = 0.021). The need for a second dose of insulin could be attributed to the slow decline in potassium levels. Hemodialysis-dependent patients experienced a greater blood glucose reduction compared to non-dependent patients, which was significant between 6 to 8 h and 12 to 24 h after admission. Patients who needed another dose of insulin experienced a slower decline in the potassium level compared to patients who only received one dose of insulin within 6 h and up to 12 h of admission (Table 5). At admission, the serum creatinine of hemodialysis-dependent patients had a 50% increase over their baseline, while it almost doubled for hemodialysis-non-dependent patients. Besides that, the serum creatinine continued its steady decline across all groups, and by day 3, hemodialysis-dependent patients’ levels were only 5–10% over their baseline, while it was over 40–50% for non-dependent patients (Table 6).

## 4. Discussion

Patients with reduced kidney function are at a higher risk of hyperkalemia, as illustrated in a recent systematic review and meta-analysis study. This was especially true in hemodialysis-dependent patients, in which hyperkalemia had more than double the prevalence compared to hemodialysis-non-dependent CKD patients [32]. Hyperkalemia treatment with insulin commonly results in hypoglycemia, which is also a common complication among patients with reduced kidney function [33,34]. Therefore, this study was conducted to evaluate the efficacy and safety of hyperkalemia treatment in patients with reduced kidney function. The primary objective was to compare the incidence of hypoglycemia after treatment with insulin between patients with reduced kidney function on hemodialysis and their counterparts (who are not hemodialysis-dependent), as well as the risk factors associated with it. 

The reduction in potassium levels was comparable to previous studies. The steepest reduction happens in the first few hours after treatment, usually averaging around 1 mmol/L based on insulin dose [35]. This was also not significantly different in patients with a low eGFR compared to patients with a normal eGFR [36,37]. Moreover, previous studies did not find a significant increase in the risk of hypoglycemia for patients with reduced kidney function, nor in hemodialysis-dependent patients in particular [13,14,15,34]. Likewise, this study found no significant difference between hemodialysis-dependent patients and non-dependent patients. On average, 18% of patients experienced hypoglycemia, which is comparable with previous studies as well. This means the standard treatment for hyperkalemia is not associated with an extra risk of hypoglycemia in patients with reduced kidney function or hemodialysis-dependent patients compared to patients with normal kidney function.

Factors associated with hypoglycemia were older age, comorbidity with heart failure, treatment with dialysis, and receiving a second dose of insulin. In this study, hypoglycemic patients were on average more than 4 years older than the rest of the patients; the same results were found in a previous study [38]. Similarly, a study found patients over 60 years old were at increased risk of hypoglycemia [34], while the opposite was observed in an older study where both groups averaged below 60 years [39]. 

A lower baseline blood glucose level is a risk factor for developing hypoglycemia, yet it was not significant in this study, contrary to previous studies. This observation might have contributed to the lower hypoglycemic events observed in the study compared to other similar studies [23,39,40,41]. This could be attributed to the overall hyperglycemic baseline average glucose level in the current study (11.3 ± 8.8 mmol/L). Although levels over 6 mmol/L were previously associated with hypoglycemia [34], baseline potassium levels were the same in both patients with or without hypoglycemic events. That being said, another study focusing on patients with reduced kidney function found that both high potassium and low blood glucose baseline levels were significantly associated with the incidence of hypoglycemic events [14]. Also, heart failure was the only comorbidity found to be a risk factor for hypoglycemia, but previous research has not studied this association. Nevertheless, the association between having diabetes mellitus as a comorbidity and hypoglycemia after hyperkalemia treatment was studied, and some results show no association (as in this study), while others show reduced risk for diabetic patients [19,38,39,42]. 

Insulin dosing strategies to prevent hypoglycemia were the focus of previous studies in which reduced doses resulted in fewer hypoglycemic episodes without significantly reducing treatment efficacy, even in patients with reduced kidney function [36,43,44]. While reduced insulin dosing—specifically 5 vs. the recommended 10 units—was associated with reduced hypoglycemic events, a study on weight-based vs. 10-unit insulin dosing showed no difference. The same study showed that a second dose of insulin is a risk factor for developing hypoglycemia, which we have similarly observed in our study [40]. The effects of other co-treatments for hyperkalemia on the risk of developing hypoglycemia were not studied. Thus, more research is needed to evaluate the association found in this study between hypoglycemia and dialysis as a co-treatment for hyperkalemia. 

Furthermore, fewer hemodialysis-dependent patients were admitted to the ICU than non-dependent patients. This could be because heart failure is significantly more prevalent in that group, as a previous study demonstrated a higher rate of ICU admission for hyperkalemia patients with either CKD or heart failure; thus, the comorbidity with either one would surely intensify the risk of admission to ICU [5]. On the other hand, more hemodialysis-dependent patients were readmitted with hyperkalemia within a month. This could be attributed to several factors, mainly that starting hemodialysis therapy increases the probability of admission for hyperkalemia, as a previous study indicated [2]. Other factors may include the severity of hyperkalemia at the time of initial treatment; inadequate adherence to treatment plans, including medication schedules, dietary restrictions, and dialysis sessions; or the rebound increase in serum potassium levels after hemodialysis [12].

This study has certain limitations that need to be acknowledged. The retrospective nature of the study introduces inherent limitations in controlling for all potential confounding factors that may have influenced the results, such as missing data—for example, the actual incidence of hypoglycemia might have been misestimated since our study excluded patients who did not have post-treatment blood glucose measurements. Also, this study was performed in a single center, for a restricted period, and with a small sample size, which poses limitations in detecting significant differences between the cohorts. However, this is a small population; thus, to have a large sample size of patients with reduced kidney function and hyperkalemia at the same time, a multicenter study needs to be conducted, which is beyond the capability of this project. Moreover, a multicenter study may reduce the effect of loss to follow-up for patients readmitted to a different center. Since this study relied on reviewing past medical records, its validity was contingent on the presence of comprehensive and precise information in the electronic medical records. Thus, any potential omission or underreporting of data could have influenced the outcomes of the study. Finally, both urinary potassium excretion and urine output were not measured during the follow-up period, which makes it challenging to differentiate the contribution of urinary elimination to the change in serum potassium levels. Also, different insulin and glucose dosing strategies were not examined. These limitations should be considered when extrapolating the results of our study to other similar populations. Lastly, further research is still warranted to address these limitations and provide a more comprehensive understanding of hyperkalemia management in patients with reduced kidney function.

## 5. Conclusions

Multiple complex factors contribute to the risk of complications with hyperkalemia treatment. Implementing adjusted management based on patients’ characteristics and the intensive monitoring of blood glucose levels for patients at risk after the administration of insulin could help address the issue of hypoglycemia following hyperkalemia treatment. Also, the underlying cause of hyperkalemia needs to be addressed to prevent readmission, particularly in hemodialysis-dependent patients. Finally, further research is needed to study the effect of comorbidities and co-treatment with dialysis on the risk of hypoglycemia and other therapy outcomes for hyperkalemia.

## Figures and Tables

**Table 1:** Baseline characteristics for patients with reduced kidney function.

Characteristic or Variable	Overall	Hemodialysis		*p*-Value *
		Non-Dependent	Dependent	
**Number of patients**	172	97	75	---
Age, years	65.2 ± 15.1	68.1 ± 13.6	61.3 ± 16.1	**0.003**
Sex				0.878
Male	86 (50.0)	48 (49.5)	38 (50.7)	
Female	86 (50.0)	49 (50.5)	37 (49.3)	
Body mass index (BMI), kg/m^2^	28.4 ± 8.5	29.5 ± 8.8	27.0 ± 7.8	0.050
**Comorbid conditions**				
Diabetes mellitus	129 (75.0)	78 (80.4)	51 (68.0)	0.062
Hypertension	146 (84.9)	81 (83.5)	65 (86.7)	0.566
Heart failure	37 (21.5)	27 (27.8)	10 (13.3)	**0.022**
Ischemic heart disease	27 (15.7)	12 (12.4)	15 (20.0)	0.173
**Laboratory values at admission**				
Blood glucose, mmol/L	11.3 ± 8.8	12.1 ± 9.8	10.3 ± 7.1	0.189
Serum creatinine, umol/L	329.0 ± 258.7	146.4 ± 94.0	565 ± 207.5	**<0.001**
Potassium, mmol/L	6.6 ± 0.7	6.5 ± 0.5	6.7 ± 0.8	**0.049**
**Home medications that may cause hyperkalemia**				
*β*-Blockers	75 (43.6)	38 (39.2)	37 (49.3)	0.183
ACE-Is	25 (14.5)	21 (21.6)	4 (5.3)	**0.003**
ARBs	23 (13.4)	21 (21.6)	2 (2.7)	**<0.001**
Potassium-sparing diuretics	15 (8.7)	13 (13.4)	2 (2.7)	**0.013**

Numbers are presented as mean ± standard deviation (SD) or number of patients with percentage (%). Abbreviations: ACE-Is: Angiotensin converting enzyme inhibitors; ARBs: Angiotensin receptor blockers. * *p*-values are from the comparison between the groups using independent sample *t*-test for continuous data and chi-square or Fisher’s exact tests for categorical data variables. Numbers in bold represent significant values.

**Table 2:** Standard management for patients with reduced kidney function admitted with hyperkalemia.

Characteristic or Variable	Overall	Hemodialysis		*p*-Value *
		Non-Dependent	Dependent	
**Number of patients**	172	97	75	
*β*-agonist	144 (83.7)	83 (85.6)	61 (81.3)	0.456
Calcium	92 (53.5)	52 (53.6)	40 (53.3)	0.971
Sodium bicarbonate	51 (29.7)	41 (42.3)	10 (13.3)	**<0.001**
Diuretics	34 (19.8)	30 (30.9)	4 (5.3)	**<0.001**
Dialysis	32 (18.6)	4 (4.1)	28 (37.3)	**<0.001**

The numbers in the table are presented with corresponding percentages from each group (column) in parentheses. * *p*-values are from the comparison between the groups using chi-square or Fisher’s exact tests as all variables in here were from categorical data. Numbers in bold represent significant values.

**Table 3:** Outcomes of hyperkalemia management.

Characteristic or Variable	Overall	Hemodialysis		*p*-Value *
		Non-Dependent	Dependent	
Hypoglycemia after insulin administration	31 (18.0)	16 (16.5)	15 (20.0)	0.553
All-cause ICU admission	26 (15.1)	21 (21.6)	5 (6.7)	**0.006**
Readmission within one-month with hyperkalemia	9 (5.2)	2 (2.1)	7 (9.3)	**0.042**
All-cause readmission within one month	30 (17.4)	17 (17.5)	13 (17.3)	0.974

Numbers are presented as patients with the outcome in the group (%), * *p*-values are from the comparison between the groups using chi-square or Fisher’s exact tests as all variables in here were for categorical data. Numbers in bold represent significant values.

**Table 4:** Factors associated with hypoglycemia in patient with reduced kidney function or hemodialysis.

Variables	Category	Hypoglycemia		OR (95% CI) *	AOR (95% CI) **
		No	Yes		
**Number of patients**		141 (82.0)	31 (18.0)	---	------
**Hemodialysis group**					
Non-dependent		81 (83.5)	16 (16.5)	Ref	------
Dependent		60 (80.0)	15 (20.0)	1.26 (0.58–2.76)	------
Age, years		64.3 ± 15.7	68.9 ± 11.3	1.02 (0.99–1.05)	**1.04 (1.01–1.08)**
Sex	Female	68 (79.1)	18 (20.9)	Ref	------
	Male	73 (84.9)	13 (15.1)	0.67 (0.30–1.48)	------
BMI, kg/m^2^		28.4 ± 8.9	28.5 ± 5.7	1.00 (0.95–1.05)	------
**Comorbid conditions**					
Diabetes mellitus	No	35 (81.4)	8 (18.6)	Ref	------
	Yes	106 (82.20	23 (17.8)	0.94 (0.39–2.31)	------
Hypertension	No	23 (88.5)	3 (11.5)	Ref	------
	Yes	118 (80.8)	28 (19.2)	1.82 (0.51–6.48)	------
Heart failure	No	115 (85.2)	20 (14.8)	Ref	Ref
	Yes	26 (70.3)	11 (29.7)	**2.43 (1.03–5.69)**	**3.92 (1.14–10.9)**
Ischemic Heart Disease	No	118 (81.4)	27 (18.6)	Ref	------
	Yes	23 (85.2)	4 (14.8)	0.76 (0.24–2.38)	0.39 (0.10–1.57)
**Laboratory values at admission**					
Blood glucose, mmol/L		11.7 ± 9.4	9.7 ± 4.9	0.97 (0.92–1.02)	0.96 (0.89–1.02)
Serum creatinine, umol/L		314.7 ± 249.4	393.7 ± 293.4	1.00 (1.00–1.00)	**1.003 (1.001–1.010)**
Potassium, mmol/L		6.5 ± 0.7	6.5 ± 0.7	0.98 (0.55–1.73)	------
**Drugs that may cause hyperkalemia**					
*β*-Blockers	No	82 (84.5)	15 (15.5)	Ref	------
	Yes	59 (78.7)	16 (21.3)	1.48 (0.68–3.23)	------
ACE-Is	No	120 (81.6)	27 (18.4)	Ref	------
	Yes	21 (84.0)	4 (16.0)	0.84 (0.27–2.67)	------
ARBs	No	122 (81.9)	27 (18.1)	Ref	------
	Yes	19 (82.6)	4 (17.4)	0.95 (0.29–3.02)	------
Potassium-sparing diuretic	No	128 (81.5)	29 (18.5)	Ref	------
	Yes	13 (86.7)	2 (13.3)	0.68 (0.14–3.17)	------
**Intervention**					
Calcium	No	66 (82.5)	14 (17.5)	Ref	------
	Yes	75 (81.5)	17 (18.5)	1.06 (0.49–2.33)	------
*β*-agonist	No	24 (85.7)	4 (14.3)	Ref	------
	Yes	117 (81.2)	27 (18.8)	1.38 (0.44–4.32)	------
Sodium bicarbonate	No	100 (82.6)	21 (17.4)	Ref	------
	Yes	41 (80.4)	10 (19.6)	1.16 (0.50–2.68)	------
Diuretics	No	113 (81.9)	25 (18.1)	Ref	------
	Yes	28 (82.4)	6 (17.6)	0.97 (0.36–2.59)	------
Dialysis	No	119 (85.0)	21 (15.0)	Ref	------
	Yes	22 (68.7)	10 (31.3)	**2.58 (1.07–6.21)**	------
Receiving a second dose of insulin	No	89 (89.0)	11 (11.0)	Ref	Ref
	Yes	52 (72.2)	31 (27.8)	**3.11 (1.38–7.00)**	**4.14 (1.68–10.21)**

Numbers are presented as mean ±standard deviation (SD) or number of patients with percentage (%). Abbreviations: BMI: Body mass index; ACE-Is: Angiotensin converting enzyme inhibitors; ARBs: Angiotensin receptor blockers. * The ORs are the crude odds ratio from the unadjusted analysis of the univariable logistic regression. ** The AORs are the adjusted odds ratio from the backward-stepwise multivariable logistic regression model. Numbers in bold represent significant values.

**Table 5:** Sub-analysis for patients who received a second dose of insulin.

Variable and Time of Follow-Up	Overall	One Dose of Insulin		Two Doses of Insulin		*p*-Value *
		Hemodialysis Non-Dependent	Hemodialysis Dependent	Hemodialysis Non-Dependent	Hemodialysis Dependent	
**Number of patients who received**						**0.021**
One dose of insulin	100/172 (58.1)	49/97 (50.5)	51/75 (68.0)	---	---	
Two doses of insulin	72/172 (41.9)	---	---	48/97 (49.5)	24/75 (32.0)	
**Dose of the repeated insulin**						0.597
Standard dose (10 units)	68/72 (94.4)	---	---	46/48 (95.8)	22/24 (91.7)	
Other doses (Less than 10 units)	4/72 (5.6)	---	---	2/48 (4.2)	2/24 (8.3)	
**The mean of blood glucose**						
On admission	11.3 ± 8.8	12.3 ± 10.3	10.0 ± 6.8	11.9 ± 9.4	10.9 ± 7.8	0.662
From 2–4 h from admission	14.4 ± 12.1	18.1 ± 14.8	10.7 ± 7.0	16.0 ± 14.6	11.4 ± 7.9	0.453
From 6–8 h	11.4 ± 7.3	12.4 ± 9	11.6 ± 7.4	11.8 ± 6.5	7.7 ± 2.6	**0.010**
From 8–12 h	10.5 ± 6.4	11.1 ± 4.9	10.9 ± 8.4	10.4 ± 5.5	8.9 ± 7.4	0.548
From 12–24 h	9.3 ± 5.5	10.1 ± 6.0	7.1 ± 4.5	11.2 ± 5.9	7.2 ± 2.8	**<0.001**
Day 2	8.9 ± 4.5	8.9 ± 3.8	7.1 ± 3.9	10.8 ± 4.6	9.2 ± 5.9	0.243
Day 3	8.8 ± 4.9	9.1 ± 4.1	7.2 ± 4.6	10.1 ± 5.3	8.9 ± 5.9	0.449
**The mean potassium level**						
On admission	6.6 ± 0.7	6.4 ± 0.6	6.6 ± 0.8	6.5 ± 0.5	6.8 ± 1.0	0.138
Within 6 h	5.6 ± 0.8	5.4 ± 0.8	5.3 ± 0.6	5.9 ± 0.5	6.0 ± 1.0	0.735
From 6–12 h	5.4 ± 0.9	5.0 ± 0.8	4.8 ± 1.0	5.9 ± 0.8	5.7 ± 0.6	0.371
From 12–24 h	5.2 ± 0.8	4.8 ± 0.8	5.1 ± 0.8	5.5 ± 0.7	5.7 ± 0.8	0.366
Day 2	4.7 ± 0.6	4.4 ± 0.6	4.7 ± 0.6	4.8 ± 0.6	4.9 ± 0.7	0.311

Numbers are presented as mean ±standard deviation (SD) or number of patients with percentage (%), * *p*-values are for the comparison between the groups of patients with two doses of insulin only. *p*-values are from the comparison between the groups using independent sample *t*-test for continuous data and chi-square or Fisher’s exact tests for categorical data variables. Numbers in bold represent significant values.

**Table 6:** Serum creatinine levels for patients who received two doses of insulin compared to patients who only received one dose.

Variable/Time of Follow-Up	Overall	Hemodialysis			
		Non-Dependent		Dependent	
		One Dose	Two Doses	One Dose	Two Doses
**Serum Creatinine, umol/L**					
Baseline	329.0 ± 258.7	151.8 ± 89.4	141.0 ± 99.1	583.1 ± 220.4	526.7 ± 175.3
On admission	533.3 ± 351.4	305.9 ± 172.0	273.0 ± 133.4	865.1 ± 289.6	813.0 ± 245.8
Day 1	449.8 ± 297.3	269.8 ± 155.1	250.0 ± 132.8	684.9 ± 271.2	719.8 ± 244.9
Day 2	405.5 ± 279.3	236.2 ± 136.1	230.8 ± 125.0	657.3 ± 254.0	646.8 ± 242.6
Day 3	384.0 ± 273.1	217.0 ± 142.1	218.0 ± 124.1	614.3 ± 280.0	581.6 ± 191.1

Numbers are presented as mean ± standard deviation (SD).

## Data Availability

The raw data supporting the conclusions of this article will be made available by the authors on request.
